# The immunomodulatory role of *withania somnifera* (L.) dunal in inflammatory diseases

**DOI:** 10.3389/fphar.2023.1084757

**Published:** 2023-02-22

**Authors:** Hamad H. Alanazi, Elyasa Elfaki

**Affiliations:** Department of Clinical Laboratory Science, College of Applied Medical Sciences-Qurayyat, Jouf University, Al Jouf, Saudi Arabia

**Keywords:** immunomodulation, immunostimulatory effects, withania somnifera, innate immunity, immune receptors, adaptive immunity

## Abstract

*Withania somnifera* (L.) Dunal (Solanaceae) (also known as Ashwagandha) is a botanical drug that has been used for centuries to treat many chronic diseases like high blood pressure, arthritis, diabetes, Alzheimer’s disease, and depression. As many botanical drugs, *w. Somnifera* possesses anti-inflammatory, antioxidant, anticarinogenic, anti-diabetic, and anti-asthmatic properties. *W. somnifera* is often compared to the ginseng plant due to its ability to reduce stress, improve cognitive functions (e.g., memory), and promote a healthy immune system. It promotes immunomodulatory effects whose function is to balance the humoral and cellular responses of the adaptive immune system. The therapeutic effect of *w. Somnifera* is attributed to active ingredients like alkaloids, steroidal lactones (such as withanolides, withaferins), and steroidal saponins. Although *w. Somnifera* is safe and highly recommended for treating various diseases, the current knowledge and understanding of its operational mechanisms are limited. One of the proposed mechanisms states that *w. Somnifera* promotes cellular-mediated immunity or initiates chemical interactions that contribute to therapeutic effects. *Withania somnifera* has been shown to play a significant role in immunological diseases by modulating several cytokines, increasing T-cell proliferation and enhancing macrophages functions. In this review, we will discuss the latest therapeutic effects of *w. Somnifera* on a number of diseases through modulating immunological markers and which specific components of *w. Somnifera* induce these therapeutic activities. We will also focus on the chemical properties in *w. Somnifera* components and their immunomodulatory role in type 2 allergic diseases where type 2 inflammation is highly imbalanced.

## 1 Introduction

The immune system deals with a spectrum of foreign antigens derived from microbes (e.g., bacteria, viruses) while maintaining normal responses to self-antigens from host tissues. Normally, invaders of the immune system are eliminated by the innate immune organs and cells, however, when innate immunity fails, adaptive immunity is initiated. Components of the innate immune system include skin, tears, low stomach pH, and body temperature while the adaptive immune system is mainly composed of T and B lymphocytes. When the innate immune system fails to clear microbes, the adaptive immune system responds in two main ways (cell-mediated and humoral) based on the type of organism. Viral and intracellular bacteria require cell-mediated immunity. In the case of microbial infection by viruses, the innate natural killer cells (NK cells), the adaptive T helper 1 (Th1) and cytotoxic CD8^+^ cells are responsible for clearing virally infected cells (Sun and Lanier, 2011). NK cells differ from CD8 cells in many ways such as antigen recognition, specificity, and memory, however, but the final aim for both types of cells is similar. On the other hand, parasitic and extracellular infections require humoral immunity which involves T helper 2 (Th2), B lymphocytes, and antibodies. Initially, when bacteria are introduced into the body, neutrophils are the first innate immune responders (Kobayashi et al., 2018). Next, antigen-presenting cells (APCs) such as dendritic cells engulf the microbe (i.e., bacteria) in a process called phagocytosis and migrate to the secondary lymphoid organs such as lymph nodes to present a small part of the microbe (antigenic peptide) to adaptive Th2 cells (Kim et al., 2006). Once Th2 is activated by the antigen peptide, it will provide the activation signal to specific B lymphocytes (that encountered the same antigen probably in a different part of the body), thus resulting in the conversion of B lymphocytes into plasma cells (known antibody producer cell) (Zhao et al., 2021). There are five types of antibodies (called immunoglobulins): IgM, IgA, IgG, IgE, and IgD (Schroeder and Cavacini, 2010). The production of antibodies is mainly based on the type of microbe, for instance, viral infections result in the production of IgG while parasites lead to the production of IgE antibodies which play important role in allergic diseases (Bell, 1996). Normally, microbial infections are eliminated and few memory CD4 and CD8 cells reside for the elimination of subsequent infections by the same microbe at later times.

Immunogenic components of microbes provoke beneficial immune responses like those seen in viral and parasitic infections; these responses are utilized for therapeutic purposes. For instance, viruses and bacteria contain immunogenic double-stranded RNA (dsRNA) structures and unmethylated Cytosine-phosphate-Guanine (CpG) motifs, respectively (Hartmann, 2017). DsRNA and CpG DNA are called adjuvants and stimulate immune responses through innate receptors like toll-like receptors (TLRs) three and nine which result in the production of type I interferon (IFN) and CD4^+^ and CD8^+^ activation (Coffman et al., 2010; She et al., 2019). Many adjuvants are used for purposes like enhancing vaccine responses or antitumor responses such as TLR2 and TLR4 ligands (Urban-Wojciuk et al., 2019). Alternatively, several studies showed that botanical medicines can be used as immunomodulators or adjuvants (Kumar et al., 2012; Shi et al., 2021). The use of natural botanical products as immunomodulators is much safer compared to conventional adjuvants because there have been fewer side effects reported when consuming them and most of these botanical products have been consumed by people for thousands of years.

*Withania somnifera* L.) Dunal (Solanaceae) or *w. Somnifera* is a botanical medicine that has been used for centuries for various purposes such as dietary supplementations and treating various diseases. The clinical use of *w. Somnifera* as an immunomodulator or adjuvant in mice and humans has yielded very favorable results (e.g., anticancerogenic, arthritis treatment) (Agarwal et al., 1999; Khan et al., 2009). Adjuvants or immunomodulators work by initiating signaling pathways mainly through immune receptors found on innate cells such as dendritic cells. TLRs are essential innate immune receptors that respond to a variety of natural and synthesized agonists (Kawasaki and Kawai, 2014). Similar to microbial components, *w. Somnifera* has been shown to modulate TLRs by inhibiting influenza A virus-induced stimulation of TLR2/4 and mRNA expression of TLR2 and TLR4 (Kashyap et al., 2020). *W. somnifera* has also been used to treat allergic diseases where type 2 inflammation is dominating (Agarwal et al., 1999).

### 1.1 Role of *w. somnifera* in type 2 inflammation

The immune system responds to a verity of foreign particles in many ways while maintaining tolerance to self or harmless particles. Generally, harmless particles do not provoke significant immune responses, however, some particles (called allergens) stimulate unwanted immune responses such as skin rash, excessive mucus production, constriction of respiratory smooth muscles, diarrhea, and others (Galli et al., 2008). The main immunoglobulin responsible for many of the allergic manifestations is IgE antibody (Galli et al., 2008). IgE antibody act as a receptor on mast cells and basophils to equip these cells for future allergen exposure (Stone et al., 2010). Upon second exposure, allergens bind to the receptor on these cells (*i.e.*, IgE) and enhance type 2 allergic inflammation (Galli et al., 2008). Type 2 allergic inflammation is characterized by Th2 and B cell activation which eventually leads to the production of IgE antibodies, an increase in eosinophils, high numbers of group 2 innate lymphoid cells (ILC2), and elevated Th2 cytokines (IL-4, IL-5, IL-13) (Galli et al., 2008).

Excessive type 2 inflammation is the driving factor for several allergic diseases including asthma, eczema, dermatitis, and several others. The incidents of allergic diseases are rapidly increasing in developed countries. More than 300 million patients worldwide are living with asthma and it is expected that the cases would reach 400 million by 2025 (Serebrisky and Wiznia, 2019). The sudden increase in allergic diseases in recent years was attributed to the reduced exposure to microbes “hygiene hypothesis”, and increased exposures to indoor allergens or environmental factors (Okada et al., 2010). It is now clear that both innate and adaptive immune responses significantly contribute to allergic responses seen in patients (Maeda et al., 2019). Initially, it was thought that the adaptive T helper (Th2) cell is the main player in type 2 inflammation, but recent studies show that innate immune cells such as ILC2 cells are important for the establishment of allergic diseases (Halim et al., 2014). Stimulating innate cells through immunomodulators or adjuvants using active concentration of 1.0 mg/kg or as low as 0.001 mg/kg intratracheally was shown to skew Th2 immune response to Th1 response resulting in attenuated allergic immunity, reduced histamine, and decreased eosinophil levels in allergic mice models (Duechs et al., 2011). One of the strategies proposed to reduce type 2 inflammation seen in asthma is the use of the *w. Somnifera* (Speers et al., 2021). In a recent study, it was shown that *w. Somnifera* reduces the levels of type 2 cytokines (e.g., IL-4, IL-13) and type 2 inflammation markers such as TNF-α and IgE induced by OVA suggesting that *w. Somnifera* has immunomodulatory properties that could be utilized to attenuate type 2 inflammation (Saggam et al., 2021). One of the current immunotherapy strategies to reduce type 2 inflammation using adjuvants is by skewing Th2 immune response into Th1 immune response. It would be interesting to know the immunotherapeutic effect of *w. Somnifera* in various allergic diseases such as atopic dermatitis and hay fever.

### 1.2 *W. somnifera* and other immunomodulators

Immunomodulators can refer to substances that modify immune responses by stimulating, suppressing, or modulating the components of the innate and adaptive immune system. Adjuvants confer immunomodulatory effects when combined with vaccines by enhancing stronger immune responses mediated by T and B cells. Adjuvants, such as alum, which has been used since ∼1930 enhance immunity through stimulating Th2 immune responses *via* dendritic cells and priming CD4^+^ and CD8^+^ cells (McKee et al., 2009). The use of adjuvants has then broadened to skewing immune responses from Th2 to Th1 such as the case in allergic diseases (Kirtland et al., 2020). Ligands of TLR3, TLR4, and TLR9 have all been used as adjuvants to skew type 2 immune responses towards Th1 responses or Regulatory T cells (Tregs) responses (Sel et al., 2007; Gunawardana and Durham, 2018). Several types of immunomodulators including adjuvants are currently under clinical trials for their use in immunotherapy in various medical conditions like cancers such as Poly (I: C), synthetic dsRNA (Sultan et al., 2020). In many cases however, the use of these adjuvants has complicated and systemic side effects making them unfavorable to use especially in serious diseases like autoimmune diseases (Saxena et al., 2019). Therefore, more studies are now exploring the use of natural plant immunomodulators as a substitute to synthetic adjuvants.

There are many fungi or natural botanical drugs that modulate the immune system in ways akin to synthetic adjuvants. For instance, *Ganoderma lucidum* stimulates the activation of immune cells such as Т cells, macrophages, and natural killer cells (Habijanic et al., 2015). *Ga*noderma lucidum also induces antitumor and inflammatory activities by secretion of interferons, interleukins, and tumor necrosis factor cytokines (Habijanic et al., 2015; Jin et al., 2016). Also, *Curcuma longa* L. induces a number of immunomodulatory properties including anti-inflammatory, antiseptic, antitumor, and antioxidative properties and most importantly antiallergic properties through inhibition of histamine production (Kurup and Barrios, 2008).

Moreover, Lentinula edodes (Shiitake) mushroom is also shown to enhance the immune system functions through increased γδ-T and NKT cells, elevated IL-4, IL-10, TNF-α, and IL-1α levels, and reduced macrophage inflammatory protein-1α/chemokine C-C ligand 3 (MIP-1α/CCL3) and inflammatory protein C-reactive protein (CRP) (Dai et al., 2015).

**FIGURE 1:**
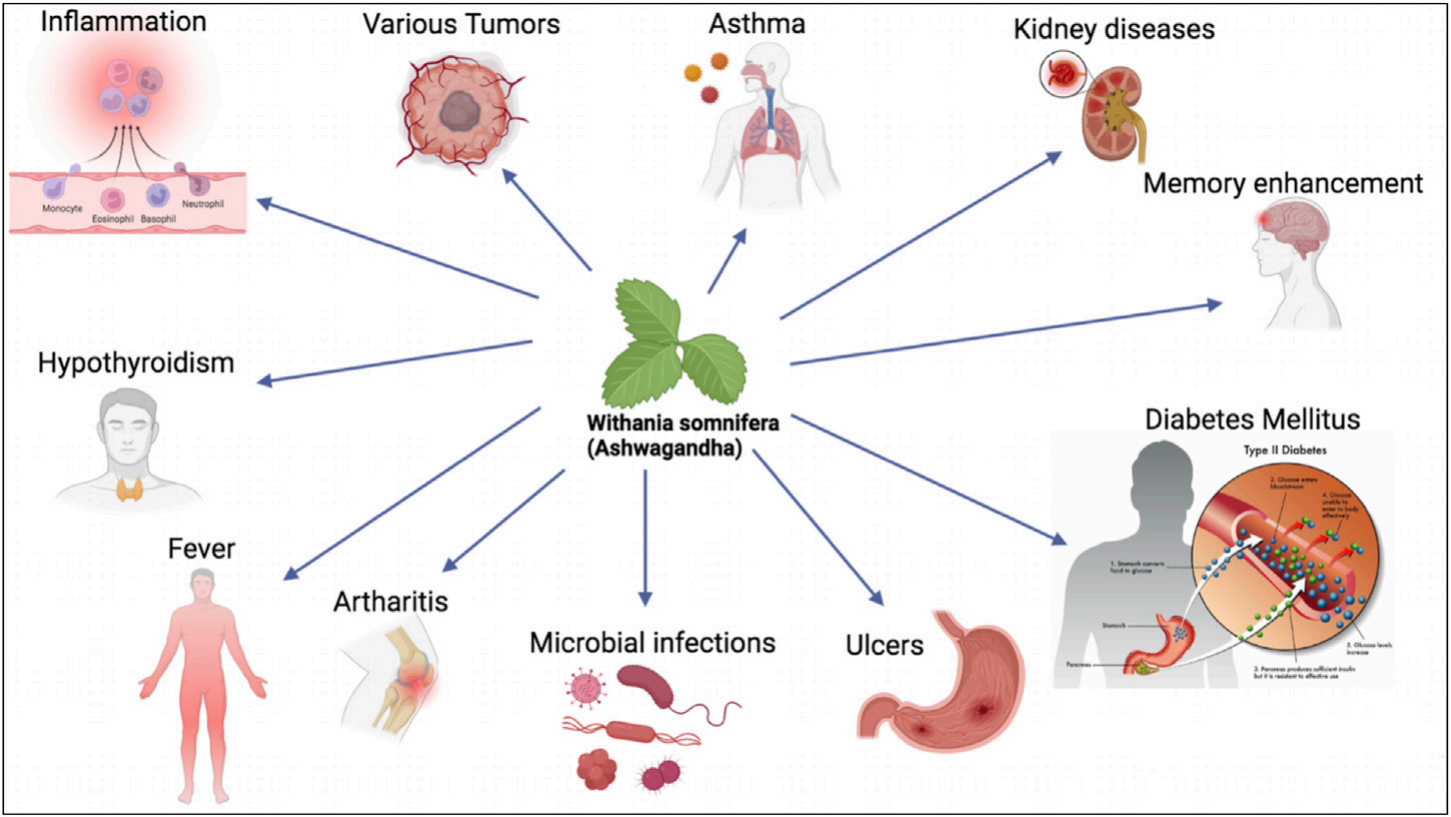
*W. somnifera* is used as botanical drug to treat various chronic diseases including asthma, kidney diseases, diabetes mellitus and others.

The immunomodulatory activities of *w. Somnifera* have been tested in several studies (Figure 1). *Withania somnifera* root powder was shown to inhibit several inflammatory mediators such as excessive complement activation, T-lymphocytes proliferation and humoral antibody response (Rasool and Varalakshmi, 2006). The role of *w. Somnifera* in attenuating Arthritis, which is an autoimmune disease characterized by increased inflammatory markers such as IL-6, IL-10 and TNF-α, has also been evaluated (Shrivastava et al., 2015). Administration of 300 mg/kg of *w. Somnifera* orally is sufficient to reduce pro inflammatory cytokines seen in arthritis such as TNF-α, IL-1β, and IL-6 *in vivo* (Khan et al., 2019). The immunomodulatory effect of *w. Somnifera* is due to presence of chemical substances such as steroids (withanolides), flavonoids and lactones (Rasool and Varalakshmi, 2006).

### 1.3 Chemical properties of *w. somnifera* compared to other immunomodulators

The steroidal lactones of group C28 known as withanolides are found in nature. Its structure consists of three cyclohexane rings, one cyclopentane ring, and four cycloalkane ring configurations (Vanden Berghe et al., 2012). Withaferin A is highly reactive due to its unsaturated lactone ring, epoxide in the B ring, and ketone-containing unsaturated A ring. For the most part, ring A’s double bond and epoxide ring are what cause cytotoxicity. The ergostane skeleton undergoes an oxidation reaction with withaferin A and associated steroidal metabolites, resulting in the formation of a six-membered delta lactone unit on the 22nd and 26th carbons. These analogs are being researched for their potential to treat inflammatory, neoplastic, autoimmune, and neurological diseases.

It has been suggested that the anti-inflammatory actions of withaferin A are due to its ability to inhibit NF-κB, AP1, and alpha-2 macroglobulin (Saleem et al., 2020). There are many withanolides that selectively inhibit COX-2 in response to inflammation (Ichikawa et al., 2006). Withaferin A’s anti-tumor capabilities were first tested in PC-3 human prostate cancer cell line xenografts in nude mice. Androgen receptor (AR)-dependent cytotoxicity was shown. In an *in vivo* pancreatic model, it suppresses tumor growth through ATP-independently inhibiting the heat shock protein 90 (HSP90) (Yu et al., 2010). In investigations using cancer cell culture, it exhibits growth-inhibitory behavior, demonstrating its cytotoxic and apoptotic capabilities. There is an increase in Mcl-1 expression *in vitro* models of breast cancer apoptosis. By covalently switching the cysteine residue in the mainly preserved alpha-helical coiled 2B domain, withaferin A can bind to the intermediate filament protein and vimentin (Bargagna-Mohan et al., 2007). Withaferin A can lead to apoptosis by causing vimentin accumulation and F-actin aggregation. In both *in vitro* and *in vivo* experiments, WA was applied to mouse sarcoma 180 (S-180) solid and ascites tumor cells. When the substance was observed using an electron microscope, it was discovered that the cells’ spindle microtubules were affected (Shohat et al., 1976). At a non-toxic concentration of about 2 μM, WA revealed a sensitizer enhancement ratio of 1.5 for *in vitro* cell death of V79 Chinese hamster cells (Devi, 1996).

Through the combination of withaferin A and the steroid lactone withanolide E, withaferin A specifically suppresses the immune system in human B-cell and T-cell as well as mice thymocytes (Shohat et al., 1978). These chemicals prevent typical B-cells and T-cells from forming the E rosette and the EAC rosette at very low concentrations. Withaferin A has specific effects on antigen recognition and the ability of both B-cells and T-cells to proliferate (Mohan et al., 2004). According to a recent study, withaferin plays a part in inhibiting Zap70 kinase activity, which is essential for human T-cell function in both sickness and health (Fazil et al., 2021).

Through the induction of Par-4, withaferin A reduces cancer cells’ capacity for invasion and their ability to move around the body (Rah et al., 2012). Withaferin stops the spread of cancer cells by preventing them from invading because the ability of cancer cells to infiltrate the extracellular matrix (ECM) is a critical determinant in the progression of cancer. Withaferin derivatives reduce the capacity of cancer cells to form colonies in a dose-dependent manner (Rah et al., 2012). Directed growth, adhesion, and migration are three biological functions that depend on actin. Withaferin A can alter the organization of the cytoskeleton by forming a covalent binding with the flexible adaptor protein annexin II and by boosting the activity of the basal F-actin cross-linking mechanism (Falsey et al., 2006).

It effectively inhibits angiogenesis (Yu et al., 2010). Chymotrypsin is inhibited by withaferin A, which has anti-angiogenic and anti-tumor properties, whilst protein kinase C is inhibited, which induces apoptosis (Sen et al., 2007). There have also been reports of withaferin A activating caspase-3.

Since withaferin A influences the immune system, it may explain why it is used as a general tonic to increase energy and ward against illness. The immunomodulatory and central nervous system (antistress, memory, and learning) effects of glycowithanolides and a combination of sitoindosides IX and X isolated from withaferin A were investigated in Swiss mice (15–25 g, 5–6 months old) and Wistar breeds albino rats (120–150 g and 250–300 g) (Nascimento et al., 2016). Both medicines significantly recruited and activated peritoneal macrophages in addition to phagocytosis and elevated lysosomal enzyme activity. Both medications significantly improved learning and memory retention in both young and old animals and drastically reduced stress in albino mice and rats (50–200 mg/kg orally). Three animal myelosuppression models using cyclophosphamide, azathioprine, or prednisolone were used to assess the root extract of withaferin A for immunomodulatory activities (Ziauddin et al., 1996). Withaferin A medication led to statistically significant increases (*p* 0.05) in hemoglobin concentration, red blood cell count, white blood cell count, platelet count, and body weight as compared to untreated control mice.

Additionally, the impact of withaferin A on the functions of macrophages obtained from mice given the carcinogen ochratoxin A was investigated (OTA) (Dhuley, 1997). Mice subjected to OTA therapy for weeks saw a considerable decline in the macrophages’ chemotactic activity. Production of TNF-α and IL-1 was also significantly decreased.

Plants and their byproducts are important sources for the creation of novel medications with special features for a range of medical uses. The widespread tendency of synthetic medications has recently given way to the use of botanical drugs (Ekor, 2014). Chemical species called free radicals and reactive oxygen species (ROS) develop in cells because of chemical reactions and metabolic activities. Free radicals can start the oxidation of biomolecules, resulting in cell damage and numerous illnesses in humans (Sharma et al., 2018). It goes without saying that antioxidant phytochemicals act as prophylactic metabolites by shielding cells from the harmful effects of free radicals and other oxidants (He et al., 2017). Alkaloids, terpenoids, polysaccharides, tannins, steroids, phenols, and flavonoids are only a few examples of the bioactive metabolites that medicinal plants produce in the form of distinctive active antioxidants that have certain biological actions against specific human diseases. The most significant adaptogen among medicinal plants is *w. Somnifera*, a treasured botanical drug known as “Rasayana” in Indian Ayurvedic medicine and a nerve tonic (Singh et al., 2011). Ashwagandha nolides are listed as active markers, along with 12 alkaloids, 40 withanolides, and sitoindosides (VII, VIII, IX, and X), according to chemical characterization of roots (Kulkarni and Dhir, 2008). Ashwagandha has been used as a vermifuge, diuretic, aphrodisiac, narcotic, astringent, and thermogenic supplement (Aggarwal et al., 2011). A tall, deciduous vine known as Tinospora sinensis (Malabar Gulbel) is known for having two novel lignan glucosides called tinoposide A and tinoposide B in its stems. When combined with other botanical drugs, these metabolites are believed to help treat muscle stiffness, lessen pain, and promote mental calmness (Haque et al., 2017). Historically, ulcerative sores, hemorrhoids, and chronic rheumatism have all been treated with the sap of the stem and leaves. The energizing fruit Phyllanthus emblica, often known as Indian gooseberry, gives the body its lost vigor and energy back. Amla is a very healthy fruit that is high in tannins, vitamin C, alkaloids, flavonoids including gallic acid, emblicanin A and B, phyllatidine, and phyllatine (Nguse et al., 2022). In addition to acting as an antiplatelet agent, vasodilator, and antiatherogenic, they have antiastringent, antidyspeptic, anticolitis, hemorrhoidal, hematuria, hepatoprotective, anti-aging, and gastroprotective effects (Hashem-Dabaghian et al., 2018). Since ancient times, Bacopa monneri, also known as “Medhya Rasayana,” or nootropic, has been used as a brain tonic to improve memory in seizure disorders and has been effective against neurological disorders. It contains bacosides A and B, brahmin, which is the main alkaloid, herpestine, and nicotine (Mannan et al., 2015). Ocimum basilicum has a group of 20 monoterpenes, triterpenes, steroids, phenols, flavonoids, and sesquiterpenes that have antihyperglycemic and hepatoprotective properties, earning it the nickname “Royal Herb” in French (Sestili et al., 2018). Its roots are classified as an anti-diabetic, galactogogue, nourishing tonic with ten different groups of steroids, such as shatavaroside, alkaloids, such as asparagine A, and specific flavonoids, and are traditionally used to treat “Vata,” hypertension, and heart disease. The miraculous botanical drug known as *asparagus racemosus*, sometimes known as the “Queen of Herbs,” helps to maintain cellular vitality (Gautam et al., 2004). Shatavari increases immunity, enhances mental abilities, helps in avoiding aging and longevity difficulties connected to stress, and promotes reproductive health (Pandey et al., 2018). The female reproductive system is nourished and supported by the uterine tonics; shatavari root tonic is typically administered to women (Pandey et al., 2018). The botanical drugs mentioned above are frequently mentioned in ancient Ayurvedic texts as a revitalizing “Rasayana” with a variety of health advantages. The potential for the presence of phytocomponents that function as cytoprotectors, anti-cancer agents, anti-inflammatory agents, immunomodulators, and immunological adjuvants led to the selection of there particular plant sections. The alleged conventional botanical drug is used in numerous recipes. The pharmacognostic and therapeutic uses of botanical drugs including ashwagandha have been demonstrated in both *in vitro* and *in vivo* models. Although there are several reports and articles on the health advantages of traditional botanical antioxidants, comprehensive combinatorial studies are still needed to increase the availability of chemical components and strengthen the body’s immune defenses.

### 1.4 Potential use for *w. somnifera* as pharmacologic agent

Withanolides are the primary biological components of *w. Somnifera* (steroidal lactones with ergostane skeleton). The withanolides have a six-membered lactone ring and a C28 steroidal nucleus with a C9 side chain. Withanolides are highly oxygenated phytochemicals, and structural differences between distinct classes of withanolides are caused by oxidation at diverse skeleton locations (Logie and Vanden Berghe, 2020). In addition, numerous classes of secondary bioactive metabolites of the plant with broad-spectrum therapeutic potential were identified and described, including withanosides, glycowithanolides, sitoindosides, alkaloids, saponins, amino acids, phenolic metabolites, flavonoids, and many others (Mishra et al., 2000; Dhar et al., 2015).

Two of the main components of ashwagandha are withaferin-A and withanolide-D, which are regarded to be largely in charge of the botanical drug’s pharmacological activity (Kashyap et al., 2022). *W. somnifera* has been the subject of in-depth toxicological investigation, and findings from several clinical research initiatives have demonstrated that the plant is safe at a variety of realistic doses. Therefore, it is reasonable to believe that the doses at which its preparations are advised on humans are similarly quite safe. To date, there have not yet been any documented botanical drug-other drugs or botanical drug-botanical drug interactions involving *w. Somnifera* in the literature. The adverse effects and long-term safety of *w. Somnifera* are currently unknown. The effects of *somnifera* could include diarrhea, vomiting, and nausea. According to a small body of research on humans, *w. Somnifera* can cause sleepiness, potentially fatal respiratory depression, low blood pressure, and irregular heartbeats (Kataria et al., 2011). The findings of studies on the chemistry and pharmacology of numerous types of *w. Somnifera* extracts derived from different portions of the plant clearly suggest that these extracts, when combined with other plants, could be used to treat and prevent a variety of illnesses and chronic diseases. The primary emphasis of this chapter is on recent and historical advancements in the chemistry and pharmacology of *w. Somnifera*.

### 1.5 Role of *W. Somnifera* in COVID-19 infection

The beneficial and inhibitory role of *w. Somnifera* has been tested in COVID-19 infection. It was shown that *w. Somnifera* disrupts the interaction between the SARS-COV-2 spike protein domain and the angiotensin-converting enzyme 2 (ACE2) receptor (Saggam et al., 2021). ACE2 receptor is considered the main receptor of entry for SARS-COV-2 into cells and is expressed in many tissues including the heart, kidneys, and lung tissues (Beyerstedt et al., 2021). In addition, certain *w. Somnifera* constituents are believed to suppress the main protease of SARS-CoV-2 which is very important for viral replication (Tripathi et al., 2021; Chakraborty et al., 2022; Shree et al., 2022). Withanoside V is one of the natural constituents of *w. Somnifera* that inhibit the main protease of SARS-CoV-2 (Tripathi et al., 2021). Moreover, several independent studies showed that *w. Somnifera* is very effective in treating COVID-19 infection probably through its ability to induce Th1 immune responses and inhibit inflammatory responses (*via* inhibiting NF-κB). *W. Somnifera* contains many other phytoconstituents such as withanolide A and B, withaferin A, withanone, and withanolides that reduce the replication and transcription of COVID-19 virus (Brahmbhatt, 2020). *W. Somnifera* induces very minimal side effects while still maintaining its therapeutic efficacy making it one of the best natural therapeutic candidates for COVID-19 infection.

## 2 Discussion

**FIGURE 2:**
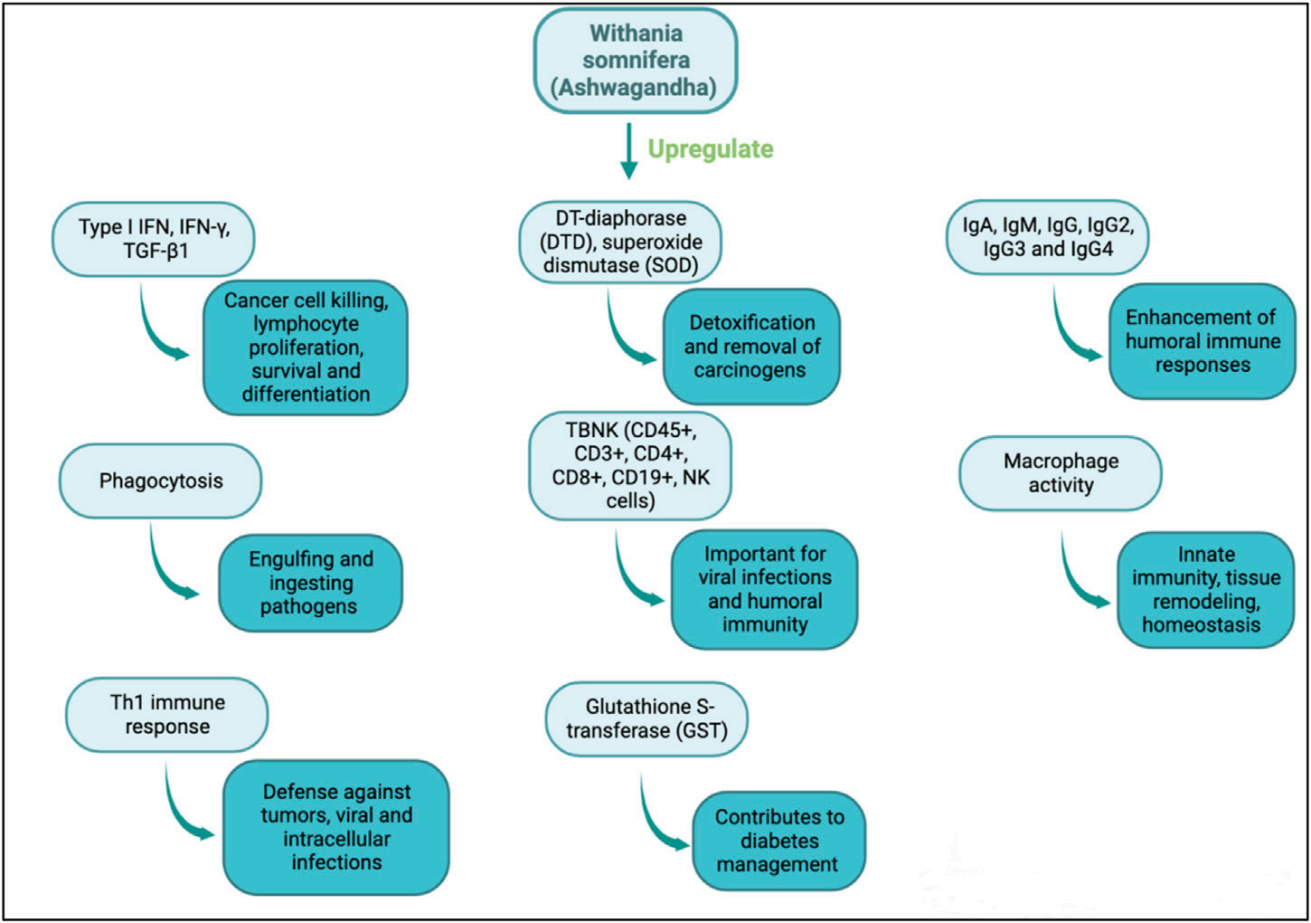
Immunomodulatory actions of *w. Somnifera* through upregulating immune mediators, cytokines, cells and enzymes.

**FIGURE 3:**
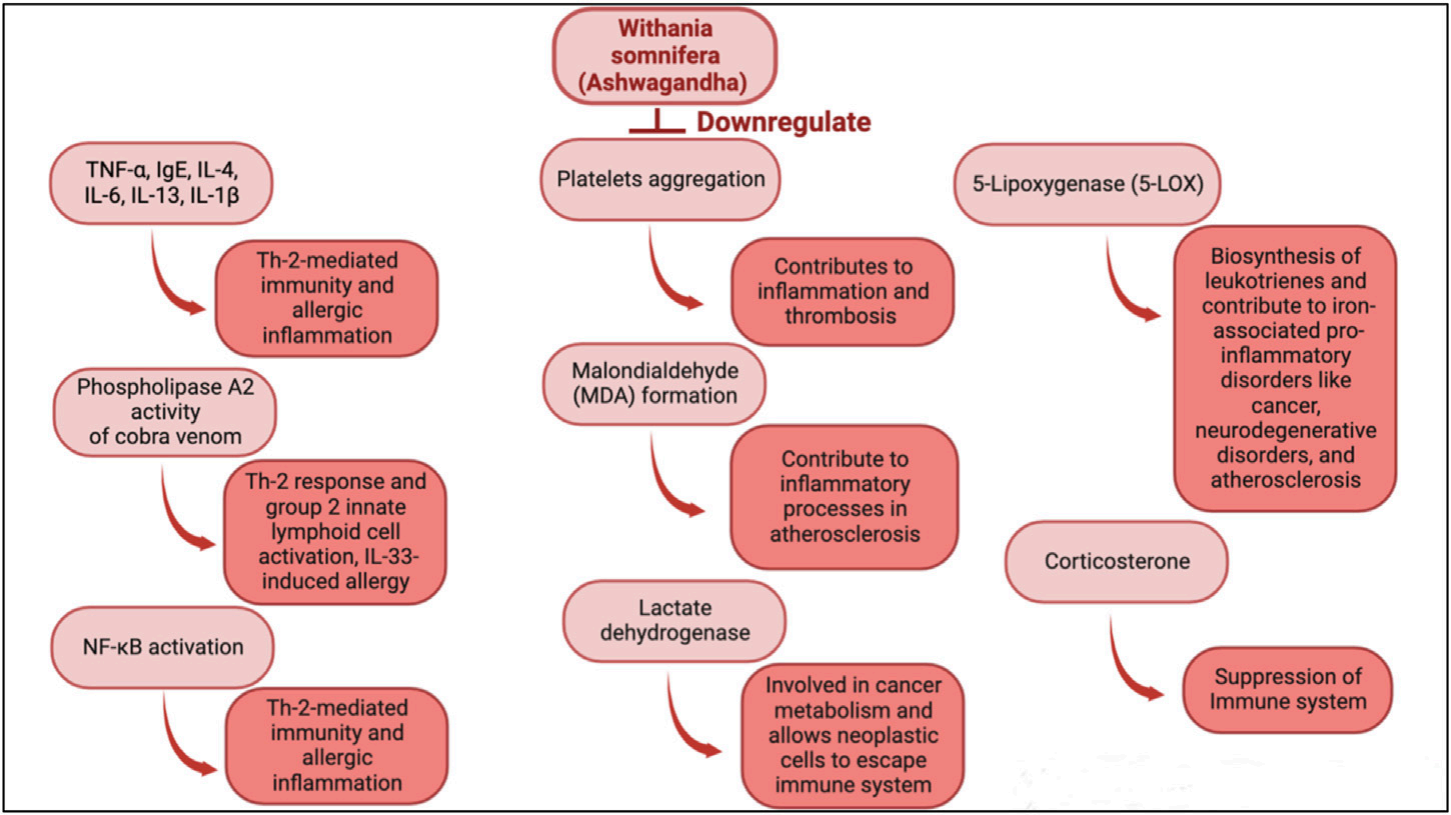
Immunomodulatory actions of *w. Somnifera* through downregulating immune mediators, cytokines, cells and enzymes.

*Withania somnifera* is a potent immune stimulator and its efficacy has been determined in multiple diseases. *Withania somnifera* induces anti-tumor, anti-stress, anti-inflammatory and immunomodulatory effects that lead to favorable and less harmful immune responses (Figure 2, Figure 3). For instance, the therapeutic effects of *w. Somnifera* on robust Th2 immune responses and airway inflammation is very promising. When administered in mice, *w. Somnifera* upregulates Th1 immune responses accompanied by an increase in CD4 and CD8 T cells, thus, balancing the Th2-Th1 immune responses (Bani et al., 2006). In asthma mice models, pre-administration of 20–80 mg/kg of withaferin A (isolated from *w. somnifera*) was effective in inhibiting airway inflammation through downregulating the expression of pro-inflammatory cytokines in the lungs (Zhao et al., 2019). Evidently, *w. Somnifera* works as an immunomodulator that mitigates the airway overresponsiveness seen in mice asthmatic models suggesting that botanical drugs, like known adjuvants, can also modulate diseases in a favorable manner (Zhao et al., 2019). Although several other botanical drugs (besides *w. somnifera*) modulate pathophysiological diseases through immune responses, the use of natural *w. Somnifera* as an adjuvant is highly encouraged. It is possible that *w. Somnifera* stimulates immune responses *via* signaling through immune receptors (e.g., TLR3, 9) found on innate cells like dendritic cells. Activating these receptors eventually results in desired immune responses.

There are two main types for adjuvants. Adjuvants that promote Th1 immune responses (cell-mediated) and adjuvants that promote Th2 immune responses (humoral) (Silva et al., 2004). In prophylactic studies, Th1 adjuvants (e.g., dsRNA) stimulate cells through innate sensors (e.g., TLR3) which shape the adaptive immunity (Chattopadhyay and Sen, 2014). In this case, Th1 adjuvants promote upregulation of interferon stimulatory gene (ISGs) response and activation of immune cells necessary for viral elimination (Chattopadhyay and Sen, 2014). Moreover, Th1 adjuvants suppress Th2 responses and can therefore be utilized to alleviate diseases where Th2 responses are hyperactive (commonly seen in allergic diseases) (Oriss et al., 1997).

## 3 Conclusion, future directions, and limitations for using *W. Somnifera* as an immunomodulator

Earlier studies on *w. Somnifera* have showed its effectiveness in treating several diseases (e.g., bacterial and viral), and allergic conditions like asthma (Alam et al., 2012). It would be intriguing to test the specific mechanisms employed by *w. Somnifera* to reduce the severity of diseases and allergic inflammation. Most of the studies investigating the therapeutic role of *w. Somnifera* (Table 1), do not fully provide complete signaling pathways starting with the initial stimulation of cells until a certain effector function of immune components is executed. It is likely that *w. Somnifera* active components (e.g., withaferin A) treat chronic diseases through stimulation of immune receptors found on innate cells (e.g., dendritic cells and macrophages). Such stimulation of these cells results in activation of downstream adaptor proteins (e.g., TRIFF or MAVS) which ultimately results in stimulation of transcription factors (e.g., IRF3) and upregulation of beneficial immune responses like Th1 immune response. Recent studies focus more on the therapeutic effects of *w. Somnifera* in many diseases including immunological diseases. In this review, we described some of the signaling mechanisms induced by *w. Somnifera* to reduce allergic conditions and diseases. For instance, in allergic conditions (where type 2 immune response is high), *w. Somnifera* provokes the production of type I IFN which suppresses type 2 immune responses. Compared to using current synthetic adjuvants which sometimes cause cytotoxicity to the host and unwanted immune responses, using a medicinal botanical drug such as *w. Somnifera* provides a safer and highly effective substitute. Defining the full strategies employed by *w. Somnifera* when used as a therapeutic agent, would empower and promote its use in many clinical trials investigating solutions for cancer, inflammatory and metabolic disorders. Further research is highly encouraged to determine the potential of *w. Somnifera as* an immunomodulator in the future.

**TABLE 1:** Immunomodulatory effects of *W. somnifera* in various diseases.

Form of WS	Immunomodulatory effects of WS	Involved diseases	References
*W. somnifera* root, leaf extract	Increase levels of immunoglobulins, IFN-gamma, CD3^+^ and CD4^+^ T-cells, T-helper 1 (Th1) cytokines and CD8^+^ T-cells	Viral diseases	Sun and Lanier (2011)
*W. somnifera* root powder	Increased Natural cells (NK) cells activity	Ovarian cancer	Sun and Lanier (2011), Kobayashi et al. (2018)
Withaferin A	Inhibition of NF-kB activity and pro-inflammatory and stress response mediators (TNFα, COX-2, and iNOS)	Inflammatory diseases	Kim et al. (2006), Zhao et al. (2021)
Withaferin A	Inhibits neutrophil adhesion, migration, and respiratory burst	Acute lung injury, acute pancreatitis, gout and apoptosis	Bell (1996), Schroeder and Cavacini (2010), Hartmann (2017)
*W. somnifera* root and leaf extracts	Maintain the normal levels for urine sugar, blood glucose, haemoglobin (Hb), glycosylated haemoglobin (HbA1C), liver glycogen, serum and tissues lipids, serum and tissues proteins, liver glucose-6-phosphatase (G6P) and serum enzymes like aspartate transaminase (AST), alanine transaminase (ALT), acid phosphatase (ACP) and alkaline phosphatase (ALP)	Hypoglycaemic and hypolipidaemic activities in Diabetes mellitus (DM)	Coffman et al. (2010)
*W. somnifera* root	Reduce serum urea, creatinine	kidney dysfunction	She et al. (2019)
Aqueous root extract of *W. somnifera*	Significantly reduce the elevated hepatotoxicity biomarkers, decrease lipid peroxidation, and enhance glutathione, catalase, glutathione reductase, and glutathione peroxidase activity	Liver injury, systematic inflammation, and liver cancer	Urban-Wojciuk et al. (2019)
Ethanol extract of *W. somnifera*	Protected against γ radiation-induced hepatotoxicity, decreased serum alanine aminotransferase, aspartate aminotransferase, alkaline phosphatase, and γ-glutamyl transpeptidase; decreased hepatic levels of malondialdehyde and total nitrate/nitrite; and increased hepatic antioxidant enzymes, including superoxide dismutase and glutathione peroxidase	Liver injury	Kumar et al. (2012)
Aqueous extract of *W. somnifera* roots	Attenuate pro inflammatory cytokines including TNF-α, IL-1β, IL-6, transcription factor NF-κB and increasing anti-inflammatory cytokine IL-10	Arthritis	Shi et al. (2021)

Like any botanical drugs and pharmaceutical drugs, there have been some adverse effects reported after *w. Somnifera* consumption. In a clinical trial, seven adult males reported feeling sleepiness, mild abdominal and joint pain after consuming ashwagandha capsule (Chauhan et al., 2022). *Withania somnifera* is still not approved by the U.S. Food and Drug Administration (FDA) and has not been used legally to treat diseases on large scale. All performed clinical trials have not been able to fully address its efficacy, mechanism, and long-term effects. In addition, there is no consensus on the adequate doses and the treatment duration for *w. Somnifera* which indicates more clinical trial research is needed. Moreover, studies investigating the potential interactions of *w. Somnifera* with other drugs are very limited.

Overall, although *w. Somnifera* induces many beneficial outcomes and reduces the severity of several diseases, there are some gaps regarding its therapeutic use as a botanical drug. Clinical studies focusing on defining the mechanisms, appropriate doses, time duration, and long-term adverse effects of *w. Somnifera* should provide knowledge that fills these gaps.
